# P-759. Comparative Effectiveness of Linezolid and Clindamycin for Necrotizing Fasciitis: Mortality and Microbiologic Outcomes in a Real-World Cohort

**DOI:** 10.1093/ofid/ofaf695.970

**Published:** 2026-01-11

**Authors:** Siddartha Guru, Paddy Ssentongo, Zinaida Perciuleac, Silvana Ribeiro Papp, Silvana Papp

**Affiliations:** Penn State Health Milton S. Hershey Medical Center, Hummelstown, PA; Penn State Health Milton S. Hershey Medical Center, Hummelstown, PA; Penn State Health Milton S. Hershey Medical Center, Hummelstown, PA; UPMC, Dover, PA

## Abstract

**Background:**

Clindamycin is commonly used as adjunctive therapy for necrotizing fasciitis due to its antitoxin properties. Linezolid, which also inhibits toxin production, may offer comparable or improved outcomes. This study evaluated the efficacy of linezolid versus clindamycin, each in combination with beta-lactam therapy, for necrotizing fasciitis

**Figure:**
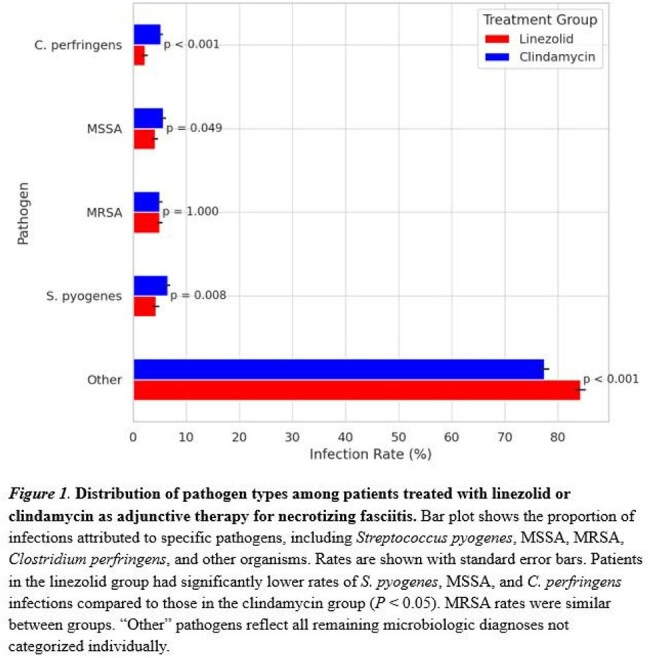

**Figure:**
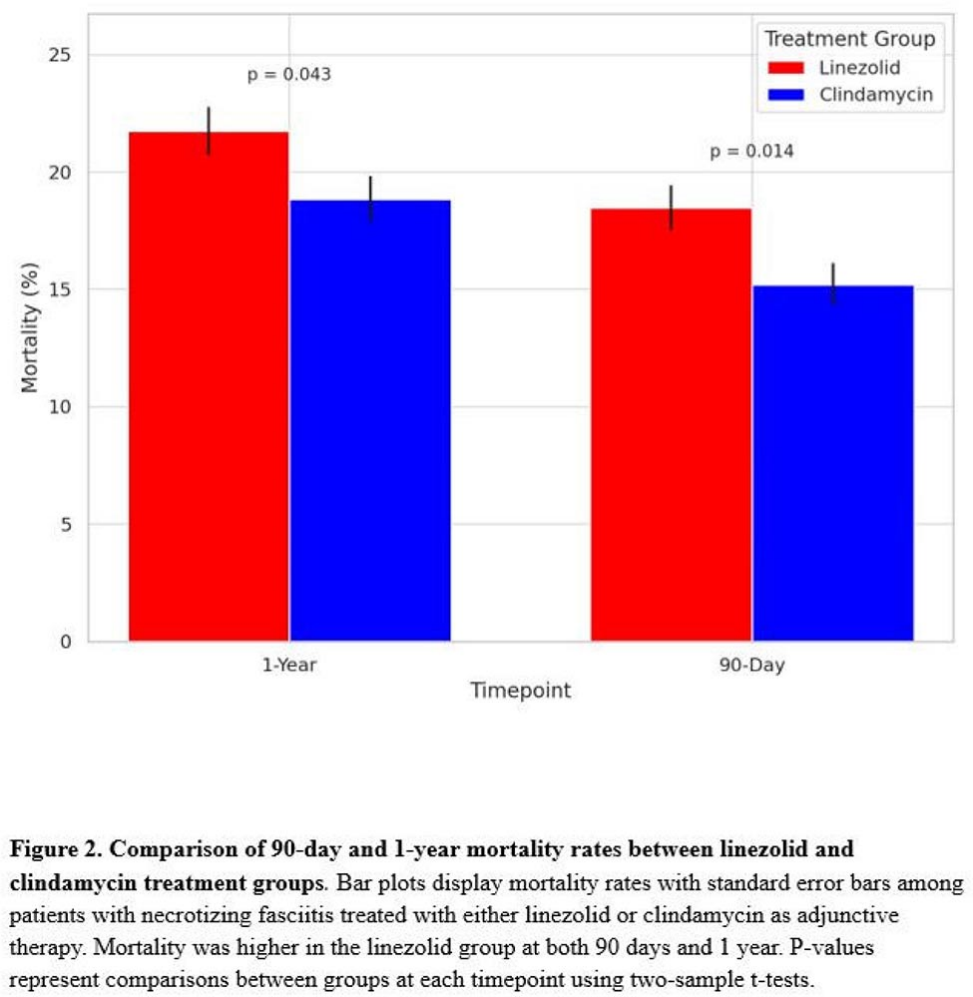

**Methods:**

We conducted a retrospective, propensity-matched cohort study using the TriNetX Global Collaborative Network. Adults aged ≥18 years with necrotizing fasciitis who received either linezolid or clindamycin as adjunctive therapy were included. Patients who received both agents were excluded. Propensity score matching was performed on age and sex. The primary outcome was 90-day mortality. Secondary outcomes included 1-year mortality, *Clostridioides difficile* infection, amputation, and thrombocytopenia.

**Figure:**
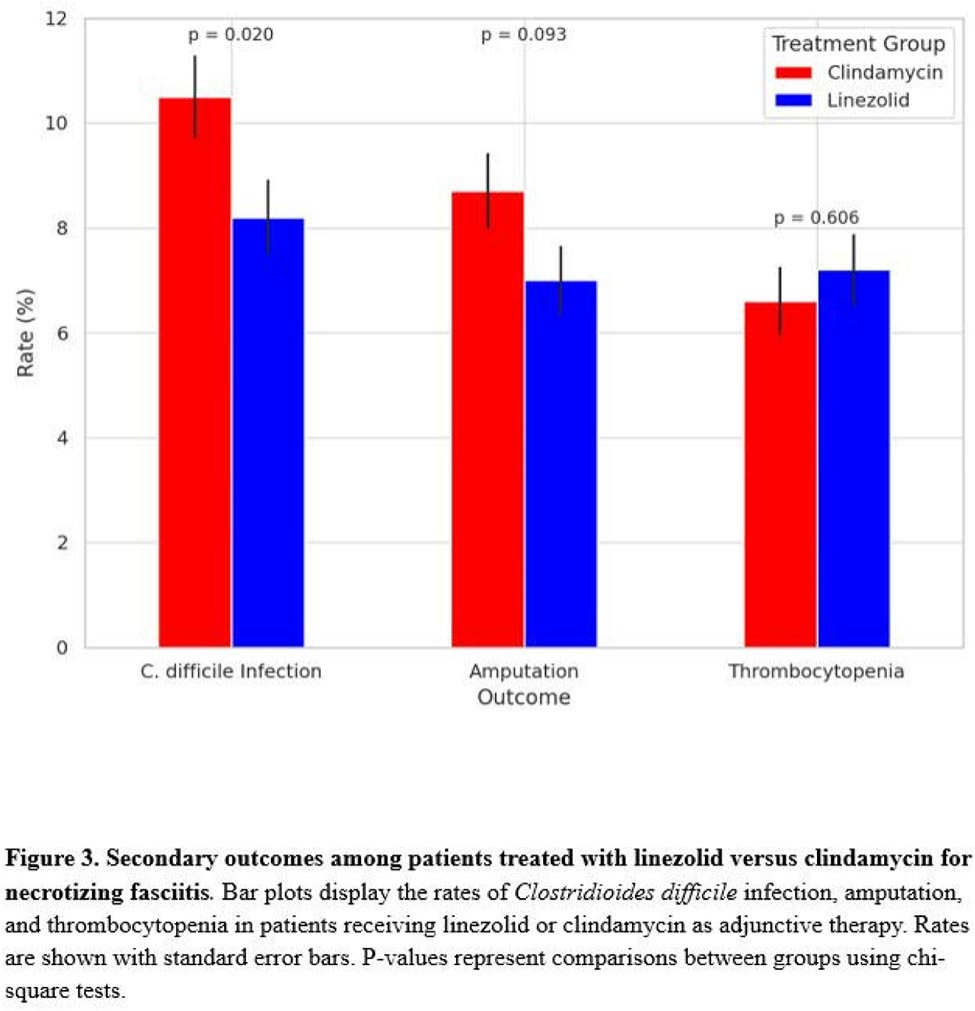

**Results:**

After matching, 1,592 patients were included in each group. Infections caused by *Streptococcus pyogenes* were less common in the linezolid group (4.5% vs. 6.7%; *P* = 0.008), as were infections due to MSSA (4.4% vs. 6.0%; *P* = 0.044) and *Clostridium perfringens* (2.3% vs. 5.4%; *P* < 0.001). MRSA infection rates were similar between groups (5.3% vs. 5.2%; *P* = 0.603, Fig 1). Infections due to other pathogens were lower in the linezolid group (84.2% vs. 77.5%; *P* < 0.001). Ninety-day mortality was higher with linezolid compared to clindamycin (18.1% vs. 15.7%; hazard ratio [HR], 1.27; 95% CI, 1.07 to 1.51; *P* = 0.032). One-year mortality was higher in the linezolid group compared to the clindamycin group (23.1% vs. 17.8%; hazard ratio [HR], 1.27; 95% CI, 1.07 to 1.51; *P* = 0.006, Fig 2). Linezolid was associated with a lower risk of *C. difficile* infection (8.2% vs. 10.5%; *P* = 0.027). Rates of amputation (7.0% vs. 8.7%; *P* = 0.080) and thrombocytopenia (7.2% vs. 6.6%; *P* = 0.526) were similar between groups (Fig 3).

**Conclusion:**

Among patients with necrotizing fasciitis, adjunctive linezolid was associated with higher mortality but lower rates of infections due to toxin-producing organisms and other pathogens compared to clindamycin. These findings highlight important differences in microbiologic and clinical outcomes that warrant further study.

**Disclosures:**

All Authors: No reported disclosures

